# Understanding the Unique Barriers and Facilitators that Affect Men’s Initiation and Retention in HIV Care: A Qualitative Study to Inform Interventions for Men Across the Treatment Cascade in Malawi

**DOI:** 10.1007/s10461-022-03909-w

**Published:** 2022-11-18

**Authors:** Kate Coursey, Khumbo Phiri, Augustine T. Choko, Pericles Kalande, Stephanie Chamberlin, Julie Hubbard, Marguerite Thorp, Risa Hoffman, Thomas J. Coates, Kathryn Dovel

**Affiliations:** 1 Department of Medicine, David Geffen School of Medicine at UCLA, 10833 Le Conte Ave 37-121, Los Angeles, CA 90095 USA; 2 Partners in Hope Medical Center, Lilongwe, Malawi; 3 Malawi-Liverpool-Wellcome Clinical Research Programme, Blantyre, Malawi; 4 Department of Health and Behavioral Sciences, University of Colorado Denver, Denver, USA; 5 Division of Infectious Diseases, David Geffen School of Medicine, University of California Los Angeles, Los Angeles, USA; 6 University of California Global Health Institute, San Francisco, USA

**Keywords:** HIV care, ART, Retention, Initiation, Sub-Saharan Africa

## Abstract

Men in sub-Saharan Africa are underrepresented in antiretroviral therapy (ART) programs. Our secondary analysis of 40 in-depth interviews with Malawian men living with HIV examined barriers and facilitators for ART initiation versus retention. Interviewees included men who never initiated or initiated ART late (initiation respondents, n = 19); and men who initiated ART but were late for an appointment (retention respondents, n = 21). Transcribed interviews were coded using deductive and inductive coding techniques and analyzed using constant comparison methods. Long wait times, frequent facility visits, and insufficient in-clinic privacy were barriers for initiation and retention. Poor knowledge of ART was primarily a barrier for initiation; unexpected travel was a barrier for retention. Key facilitators for initiation and retention included previous positive experiences with health facilities. Having examples of successful men using ART primarily facilitated initiation; support from spouses and male peers facilitated retention. Results may inform interventions to increase men’s engagement in ART services.

## Introduction

Men are underrepresented in antiretroviral therapy (ART) programs in sub-Saharan Africa, with lower rates of both ART initiation and retention as compared to women [1–4]. In Malawi, men represent 39% of new adult ART initiates [5], and one study found that male ART clients were more likely than female ART clients to be lost to follow-up for up to eight years after ART initiation [6]. Men who fail to utilize ART services need targeted, tailored programs to improve their engagement with HIV care, yet few interventions are designed to reach disengaged male clients across the treatment cascade [7, 8]. To our knowledge, no study to date has directly compared and contrasted barriers and facilitators across ART initiation and retention (i.e., the treatment cascade). This is important because such comparisons can identify interventions that reach across the cascade and what discrete strategies are needed at each stage of the continuum (if any).

Barriers and facilitators may be similar across the continuum of care, such as lack of privacy during ART appointments [9, 10], fear of unwanted disclosure, and fear of HIV-related stigma [11–16]. Lack of privacy may be experienced (or expected) by men who never initiate ART and those who have been in care for an extended period of time. If these same men also have fear of HIV-related stigma, the lack of privacy may lead them to avoid ART services altogether. At the same time, initiation and retention may present distinct challenges for men that require different interventions. Misconceptions regarding ART may disproportionally impede ART initiation, such as fear of side effects, poor knowledge about the benefits of ART, and feeling healthy at the time of diagnosis [17–21]. Logistical barriers such as transportation costs, distance to health facilities, long wait times, and provider absences [14, 22, 23] may have greater influence on retention since men are likely to experience them throughout their engagement in HIV care.

A deeper understanding of the specific barriers and facilitators experienced by men at various stages of treatment is needed to develop interventions that reach men across multiple ART touchpoints. We conducted a secondary analysis of in-depth interviews with two groups of men who failed to optimally engage in ART services (either at initiation or retention) in Malawi and compared barriers and facilitators across the care continuum.

## Methods

### Study Setting, Sites, and Participants

We conducted a secondary analysis of in-depth interviews collected between 2016 and 2017 as part of a larger study that examined the impact of universal treatment policies in Malawi. Details about the parent study have been published elsewhere [24, 25]. The parent study aimed to understand barriers to and facilitators of ART engagement under universal treatment policies in Malawi. Ten mid-size health facilities in rural central and southern Malawi were purposively selected for the parent study.

Men were included in the parent study if they met the following eligibility criteria: (1) ≥ 18 years of age; (2) sought HIV services at a participating health facility; and (3) either did not initiate ART ≥ 14 days after testing HIV-positive *or* were ≥ 14 days late for an ART appointment within the first 12 months after ART initiation but eventually re-engaged in care. For this secondary analysis, we analyzed in-depth interviews with men from two groups of the treatment cascade: Group 1—those who never initiated ART or initiated ≥ 14 days after testing HIV-positive, henceforth referred to as initiation respondents who completed interviews regarding experiences with initiation; or Group 2—those who were ≥ 14 days late for an ART appointment but eventually returned to care, henceforth referred to as retention respondents who completed interviews regarding experiences with retention. All initiation respondents were offered and refused same-day ART initiation at the time of receiving a positive HIV test result.

### Respondent Recruitment and Data Collection

The parent study used purposive sampling, sequentially recruiting eligible individuals who were identified by healthcare workers or study staff. Individuals who never initiated ART were traced by community healthcare workers in order to encourage them to initiate ART. Upon successful tracing, healthcare workers also invited individuals to participate in the study and referred interested clients to study staff. Individuals who initiated ART were identified as potentially eligible for an interview through medical chart reviews. Study staff recruited eligible clients as they waited for routine ART services at participating health facilities. Interviews were conducted in private, confidential places, physically separated from main waiting areas.

In-depth interviews were conducted by experienced Malawian qualitative researchers. All interviews followed a pre-developed interview guide specific to ART initiation or retention. Both guides included basic modules around socio-demographics, individuals’ experience with HIV and ART services, reasons for not initiating ART or missing ART appointments, and facilitators to ART initiation or returning to care, although questions were not identical between initiation and retention respondents. Specific probes around barriers and facilitators at varying levels of the socio-ecological model (individual, intrapersonal, community, facility) were also included. Interviews were audio recorded and lasted between 40 and 80 min. Audio recordings were transcribed, translated to English, and analyzed in Atlas.ti v8 [26].

### Data Analysis

For this secondary analysis, KC, PK, and KD developed an initial codebook based on existing literature on barriers and facilitators to ART care for men and informed by the socio-ecological model [17, 27–29]. KC and PK piloted the codebook by separately coding the same four interviews and compared coding with KD, resolving any discrepancies and adding codes iteratively as needed. Analyses focused specifically on barriers and facilitators to ART engagement among men, as self-reported by men themselves. Thematic codes were stratified by the timepoint to which the respondent referred (either ART initiation or retention); the vast majority of initiation codes came from interviews with initiation respondents and vice versa. All data were analyzed using constant comparison method [30], comparing differences and similarities between themes. Analysis of themes was done separately for the following categories: barriers to initiation, barriers to retention, facilitators to initiation, and facilitators to retention. Themes that were most commonly or commonly mentioned were considered to be the major themes for their respective categories. These data are also presented visually to enable comparison between initiation and retention responses.

### Ethical Considerations

The study was approved by the National Health Science Research Committee (NHSRC) in Malawi and by the Institutional Review Board at the University of California Los Angeles. All participants gave oral consent prior to completing an interview. No identifying information was collected.

## Results

Forty in-depth interviews with men were included in the analysis: 19 initiation interviews (men who either never initiated or initiated late) and 21 retention interviews (men who initiated ART but were late for a follow-up ART refill appointment). Participants’ ages ranged from 21 to 59 years with a mean age of 35 years, 87% were married, and 89% had children. Among initiation interview respondents, 13/19 never initiated ART (see Table 1). Throughout the results section, we highlight similarities and differences in barriers and facilitators to ART engagement for men across the treatment cascade.

**Table 1:** Demographics

Demographics	Initiation interview respondents (n = 19)	Retention interview respondents (n = 21)
Age, mean (range)	34 (26–50, 2 unknown)	35 (21–59)
Married, n (%)	17 (90%)	17 (85%, 1 unknown)
Children, n (%)	15 (88%, 2 unknown)	19 (91%)
Ever initiated ART, n (%)	6 (32%)	21 (100%)

### Barriers

**Fig. 1:**
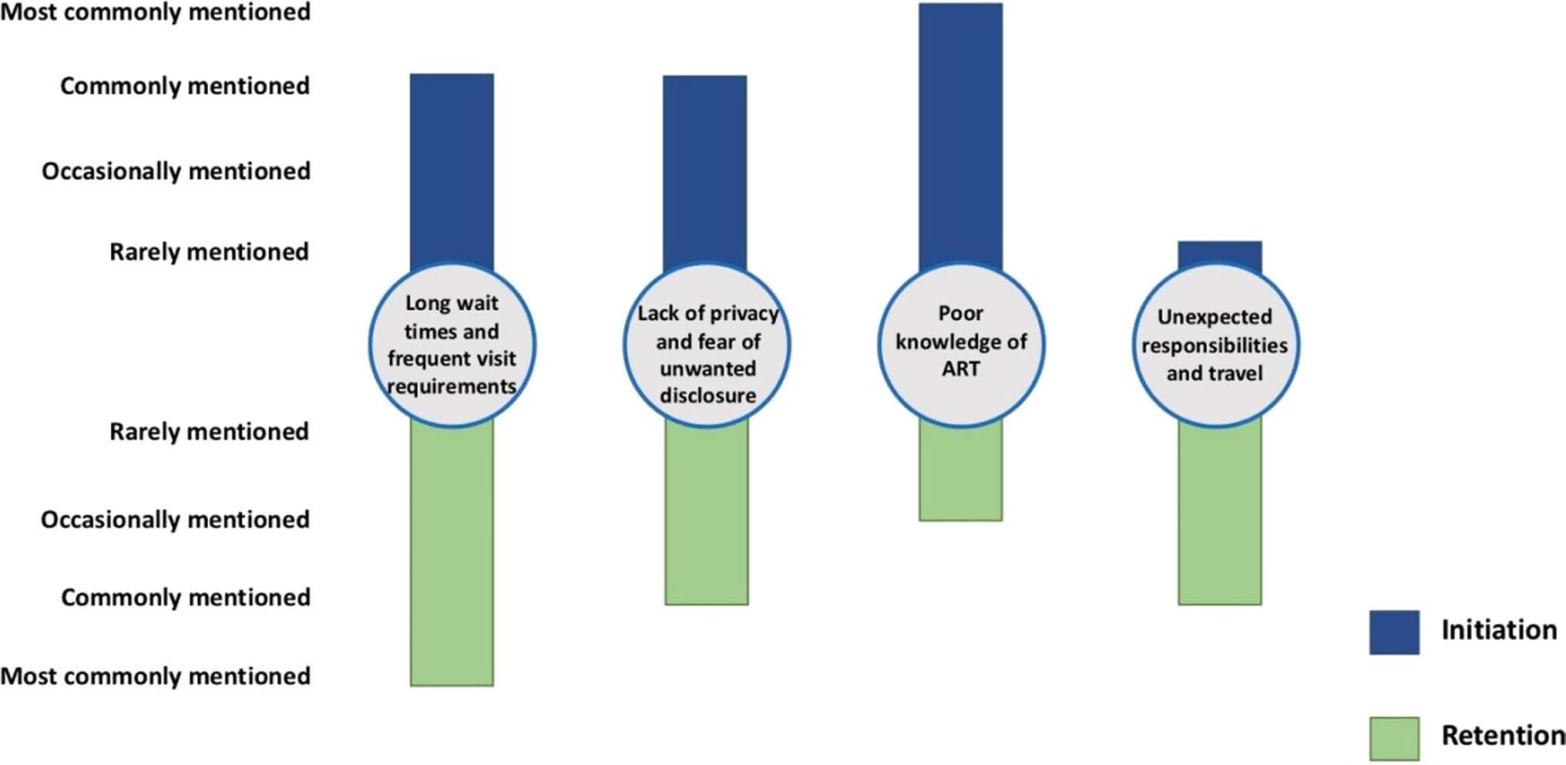
Barriers to ART engagement for men’s ART initiation and retention in Malawi

Figure 1 shows four key barriers to men’s ART engagement across the care continuum. Time requirements related to long wait times and frequent facility visits, as well as lack of privacy at facility visits, were major barriers for both ART initiation and retention. Lack of ART knowledge was the most commonly mentioned barrier for initiation, while unexpected travel was commonly mentioned for retention. Among initiation respondents, all three major barriers to initiation occurred relatively more often among respondents who had never initiated ART compared to those who initiated ART late. We discuss each barrier below in detail.

#### Long Wait Times and Frequent Visit Requirements

Extensive time commitments required to access ART services was seen as a major barrier for initiation and retention. Facilities had inflexible service hours, which required men to attend facilities during hours that conflicted with their work schedules. Both initiation and retention respondents often prioritized income-earning activities over facility visits because they were worried about being able to provide financially for family and other dependents.

*“I run a butchery, and I have to go into the villages to find goats to buy…it would be easier to get my ART if the service is faster, but if it is slow, then it is very difficult for me to go [to the clinic], and I could even stop altogether…I live on my business, so me and my family could starve.”* – Initiation respondent (late initiate), age 34

Retention respondents occasionally had to make repeated trips to the health facility in order to successfully access ART services, compounding the burden of transportation costs to get to health facilities. Repeat visits were usually required due to unavailable or overburdened facility staff.

*“Sometimes, they send us back because the doctor is at home, or has gone for some training and has carried the keys for the room where the ART is…due to scarcity of transportation fare, I spent two days without coming back here.”* – Retention respondent, age 27

#### Lack of Privacy and Fear of Unwanted Disclosure

Fear of unwanted disclosure due to limited privacy at ART clinics was a commonly mentioned barrier for both initiation and retention. Perceived lack of privacy was influenced by the physical structure of clinics—in all participating study facilities, separate buildings or corridors were dedicated to ART services, and several facilities had days dedicated only to ART services. As a result, being seen at the ART clinic—or at the facility on an ART clinic day—automatically raised suspicion regarding one’s HIV status.

*“…I asked [the hospital staff], “Where do you hold the lessons?” They just said, “Right here…” That got me worried [that] everyone is going to know that “Oh, that group standing there is HIV positive.””* – Initiation respondent (non-initiate), age 26

*“All those on ART come here…and start receiving their allocations one after the other, then privacy is compromised…testing is done secretly, so why then is ART allocation public?”* – Initiation respondent (non-initiate), age 26

Among initiation respondents, non-initiates specifically expressed concerns about the consequences of unwanted HIV status disclosure on their personal and professional lives. These concerns included being blamed for bringing HIV into the home, as well as fear of losing work opportunities due to stigma.

*“I have not disclosed to anyone…There are so many young men here who can [gossip]…If the woman hears [about my HIV status] from other people, then things can get disturbed…[it] can make the woman think I have brought a burden on her life.”* – Initiation respondent (non-initiate), age 33

*“If there is any challenge then it is my job. I will be in serious trouble [if my boss finds out that I am taking ART]. I will be surely fired.”* – Initiation respondent (non-initiate), age 48

Respondents discussing retention were primarily concerned that status disclosure would lead them to experience cruel treatment and stigma at the hands of community members. One retention respondent reported that stigma led him to stop taking ART.

*“…some people were not treating me well…they were insulting me to say, ‘You are going to die anytime now since you are taking [ART].’ So I said it is better for me to just stop taking medicine and die.”* – Retention respondent, age 40

#### Poor Knowledge of ART

Poor knowledge about ART was the most commonly mentioned barrier for ART initiation, but was mentioned less frequently for retention. Many respondents were reluctant to initiate treatment due to inaccurate beliefs about side effects and fear of treatment fatigue. In many cases, men had a misconception that once an individual initiates ART, missing even a single dose could lead to severe health consequences. Men were concerned about their ability to take ART consistently every day, and several respondents indicated that it was better to postpone initiation entirely rather than risk taking ART in an inconsistent manner.

*“Because ART pills are not the kind of pills which one can take now and maybe take again after three or four days…if I will be skipping days on my treatment…the virus will take advantage and attack the body…it is better that I do not even take them in the first place.”* – Initiation respondent (non-initiate), age 26

Initiation respondents were unaware about the benefits of early initiation, resulting in little motivation to start ART while still healthy. Many respondents believed that initiating ART too early could negatively impact their health; a few men refused ART until their health worsened.

*“[ART] can make a patient faint, especially if I am to take them while I am still this strong…So I am worried that starting treatment at such any early stage could cause serious problems for me.”* – Initiation respondent (non-initiate), age 48

#### Unexpected Responsibilities and Travel

Last minute, unexpected travel that conflicted with ART appointments—primarily due to funerals and work obligations—was a commonly mentioned barrier for retention, but rarely mentioned for initiation. Yet few respondents were aware that they could access treatment at other health facilities in Malawi when traveling in order to avoid missed doses.

*“I traveled to Ntcheu to look for Irish potato, and my younger brother wasn’t home, he was at school, so I couldn’t send anyone [to the facility]…when I came back to the hospital, I explained it to the doctor, that sorry I have done myself wrong because I didn’t come this month to get my drugs…the doctor told me that [I could get ART from any public hospital while traveling]…I didn’t know.”* – Retention respondent, age 22

### Facilitators

**Fig. 2:**
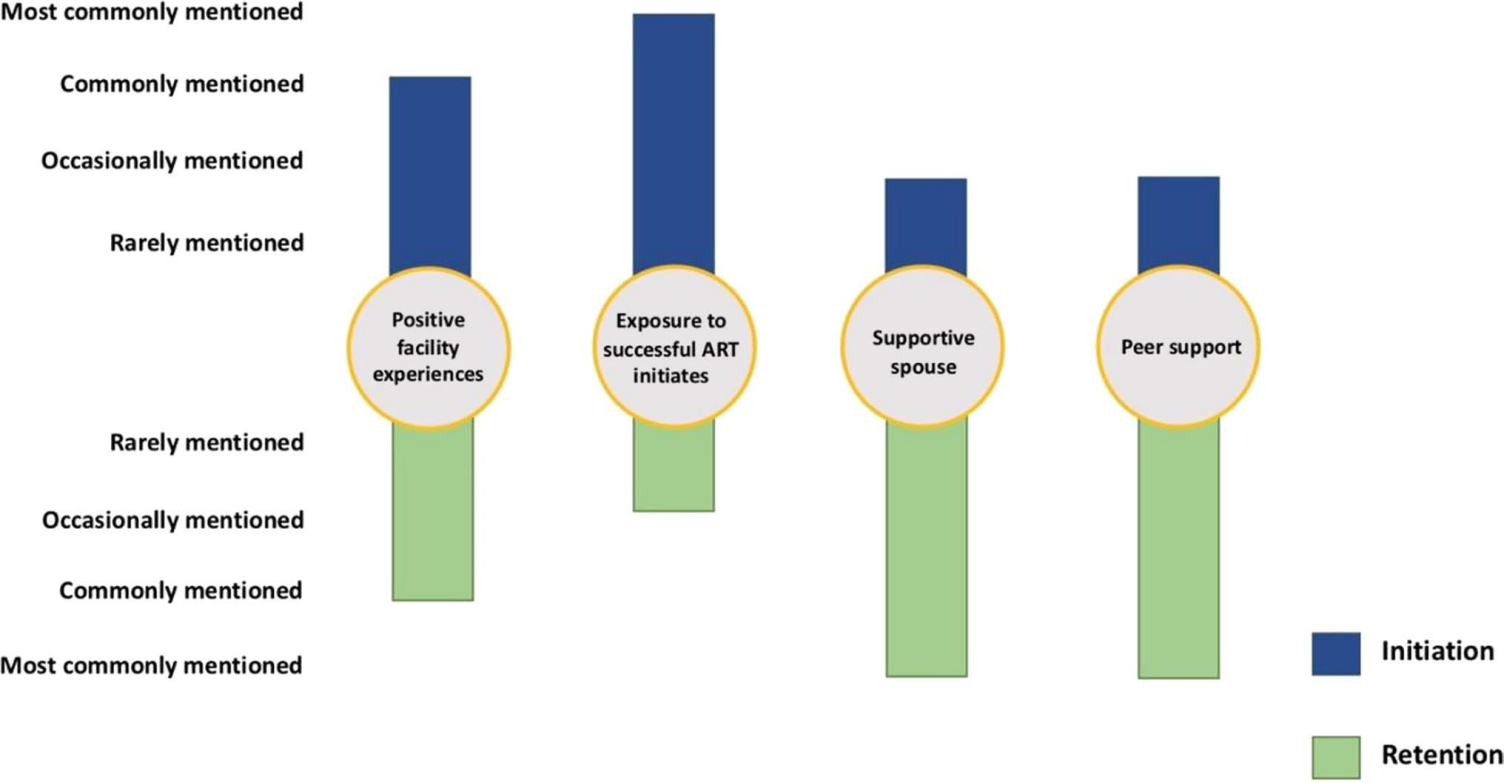
Facilitators to ART engagement for men’s ART initiation and retention in Malawi

Figure 2 shows four key facilitators to men’s ART engagement across the care continuum. Positive experiences receiving ART services at health facilities was a commonly mentioned facilitator for both initiation and retention. Exposure to someone else who was successfully taking ART in the community was the most commonly mentioned facilitator for initiation, while having support from spouses and, importantly, peers were the two most commonly mentioned facilitators for retention, reported by an equal number of respondents.

#### Positive Facility Experiences

Previous positive experiences with health facilities encouraged respondents to both start and remain in HIV care programs. For initiation, access to client-centered and detailed HIV counseling (either during HIV diagnosis or during other non-HIV visits) was key to creating these positive experiences.

*“The medical officer is there to enlighten you what happens when you delay [initiating ART] … the medical officer enlightened me on the disadvantages of delaying as well as the advantages of not delaying.”* – Initiation respondent (late initiate), age 26

For retention, having empathetic and understanding providers may have increased men’s comfort when returning to care after missing appointments.

*“Here at this hospital there is [Nurse X] … All I see is that when I come here, they welcome me well … that’s what makes me free. Plus they show a happy face to everyone.”* – Retention respondent, age 34

A few retention respondents formed social connections with fellow ART clients when attending routine facility visits. These relationships were identified as a source of support, encouragement, and advice, thus promoting an overall positive facility experience.

*“We do discuss [concerns and questions] with fellow [ART] patients. When we get here, while the doctors are late, we do discuss…sometimes I speak in a group to the extent that people laugh. I am comfortable asking my friends and discussing such things.”* – Retention respondent, age 46

#### Exposure to Successful ART Initiates

Men who successfully took ART served as powerful examples for respondents, even if the respondent did not have a personal relationship with the man. Similarly, seeing negative examples of men who became severely ill due to delaying ART was also a motivation. This finding was the most commonly mentioned facilitator for initiation, but also emerged for retention as well. High energy levels, physical strength, good physical appearance, and a robust immune system were health outcomes that men desired for themselves. When men witnessed these benefits in other men on ART, and saw the resulting benefits of financial productivity and a longer life, they were encouraged to pursue ART themselves.

*“…the doctor told me that they had been on ART treatment for eleven years and had three children. The doctor was not even looking sick or unhealthy in any way…And so that changed my mind into taking ART.”* – Initiation respondent (late initiate), age 34

*“Some of these people that you see whose bodies are fit, means that they are using pills daily. They are not missing…I get encouraged in the sense that I admire someone who is taking their pills and their body is very fit…I, too, would like to be like them.”* – Retention respondent, age 59

#### Supportive Spouse

Support from spouses was most commonly mentioned as a facilitator for retention, and less frequently for initiation. Spouses facilitated initiation by providing emotional support in the aftermath of an HIV diagnosis. For retention, spouses provided logistical support for daily medication adherence and routine attendance to ART clinics. Spouses would remind men to attend facilities and would frequently attend the ART clinic in the man’s place so that the man did not have to stop working.

*“… because I had the wife at the hospital, she was encouraging me to say we should just be bold to follow what the doctors tell us. So little by little I started to calm down.”* – Initiation respondent (non-initiate), age 26

*“Sometimes I decide to take a nap while the wife is cooking. When I wake up, she reminds me, ‘You should take your medicine’… I am able to remind her as well… I remind her because she is my friend…whenever I am coming here [ART clinic], I make sure to take my health passport and hers as well to collect her medicine.”* – Retention respondent, age 40

#### Peer Support

Peer support from other men was mentioned occasionally as a facilitator for initiation, and most commonly for retention. Several initiation respondents relied on emotional support from a close male relative after receiving a positive diagnosis.

*“[I told my brother about my diagnosis] because he is the only person whom I have ever been open with on each and every matter…if I had been very healthy or very ill, I would still have disclosed my status to him…even if I have started taking ART, he is the one person who will be able to encourage me…to say, “Hey man, you need to take your pills now…””* – Initiation respondent (non-initiate), age 26

Retention respondents similarly mentioned social support from male relatives, but they also frequently relied on social support from unrelated male friends (which was not mentioned when discussing initiation). Multiple retention respondents discussed having male friends who provided emotional support and served as confidants for taking ART, often reassuring respondents that they appeared strong and healthy due to their continued use of treatment. Some men described a sense of camaraderie amongst other men who were also living with HIV, indicating that spending time with these friends and taking medication together encouraged them to take ART.

*“There are some friends with whom I play football, and they bring their medication to the grounds. When the sun sets, they take their medication as they leave the football ground on their way home. So we encourage each other…[they encourage me] very much, and it is something that is not shameful.”* – Retention respondent, age 32

## Discussion

Our qualitative study in Malawi found numerous similarities in the barriers and facilitators experienced by men who struggled with ART engagement across the treatment cascade. Long wait times and frequent facility visit requirements, as well as lack of privacy and fear of unwanted disclosure, were major barriers for both men who struggled with timely ART initiation and those who struggled with retention. Positive experiences at health facilities facilitated care across the continuum. Several divergences appeared: poor ART knowledge was the most common barrier to initiation but less frequently reported for retention, while unexpected or prolonged travel was primarily mentioned as a barrier to retention. Exposure to successful, respected men who used ART was the most commonly mentioned facilitator for ART initiation, while social support from spouses and male peers facilitated retention. Findings can inform comprehensive interventions for men across the care continuum, highlighting the need to address time requirements, privacy concerns, and promote positive facility experiences at all ART touchpoints. Client-centered, detailed counseling should be provided immediately after receiving a positive diagnosis (rather than waiting until initiation), and peer role models and peer support should be promoted throughout the continuum.

Long wait times and frequent facility visits are known barriers to HIV care [31, 32]. Monthly ART visits are required by Ministry of Health (MOH) guidelines in the first six months after initiation. The burden of care-seeking during this timeframe can serve as a deterrent for ART initiation (due to anticipated burden of care) and early retention (due to experienced burden of care). Time-related barriers may be compounded by lack of privacy at facilities (and therefore fear of unwanted disclosure), because men who spend numerous hours at an ART clinic every month are more likely to be seen by community members waiting for HIV care. Integrating ART services within primary care could address privacy concerns for both initiation and retention [33–35], but integrated services can be logistically challenging to implement [36, 37]. For retention, multi-month dispensing (MMD) can reduce the number of facility visits required and therefore the burden of care, as well as reducing the risk of unwanted disclosure since men attend ART clinics less frequently [38–40]. MMD could also promote ART initiation amongst those who are otherwise healthy by decreasing perceived time required to access care (for example, offering three months of ART upon initiation). Further research is needed on the clinical safety and provider acceptability of rapid MMD for new initiates.

Positive facility experiences facilitated care across the continuum, even if it was simply positive social experiences with other clients. Other literature in the region has highlighted that men may feel out of place at health facilities [27, 31, 41], which are often centered around women’s and children’s health [42, 43]. This did not emerge as an explicitly stated theme in our data, however the implicit identification of health facilities as the domain of women and children may partially explain the importance of positive facility experiences for promoting men’s engagement in ART services [31]. Client-centered care, waiting spaces that encourage discussion and socialization among clients, and male-tailored services could enhance positive experiences at facilities; these positive experiences may increase men’s attendance at HIV services [3, 31]. Given that many patients feel healthy at the time of diagnosis, friendly interactions at ART clinics (either with facility staff or fellow clients) may be one of the few immediate benefits associated with early ART initiation [44, 45].

Examples of successful men who use ART and live “normal,” healthy lives (i.e. role model) was a facilitator for both initiation and retention, but was particularly essential for initiation. Interestingly, men did not have to know the role model—simply hearing examples of successful men was enough. Incorporating role models or male-specific examples into counseling materials may improve men’s confidence in their ability to achieve both engagement in ART services and income-generation, promoting initiation. In contrast, peer support was the most commonly mentioned facilitator for retention. Men in the retention group deeply valued support from male friends and family members. Very little research has highlighted the role of other men to support men’s ART engagement. Interventions should consider facilitating peer support through providing male-only support groups [27, 43, 46], offering one-on-one male mentorship [47], or supporting men to disclose to at least one male friend or family member.

Misconceptions about the benefits of early ART was seen as a major barrier for ART initiation (but not retention). In our study, initiation respondents referenced old HIV policies whereby individuals living with HIV could not initiate ART until their CD4 count was low enough to be eligible for treatment [48], suggesting that starting ART too early could be detrimental to their health. Numerous men also believed that missing even one dose of ART could have negative health consequences, leading several men to avoid initiation entirely rather than risk treatment fatigue and missing doses. These misconceptions should not be surprising, as legacies of old guidelines continue to influence community perceptions long after guidelines are changed [18]. Community sensitization on the benefits of early treatment, and having self-compassion if someone misses an ART dose, should be provided. Detailed counseling is particularly important at the time of diagnosis given rapidly changing ART programs and the fact that community perceptions do not shift as quickly as policies [18, 27, 49].

This study has several limitations. First, interviews were conducted in both facility and community settings. Facility-based interviews may introduce social desirability bias; however, interviews were conducted by non-facility staff, and we emphasized that all information was confidential and did not affect their access to health services. Second, we did not interview individuals who successfully initiated and remained in care without interruptions, nor those who missed an appointment and never returned to care, thus limiting our ability to compare barriers and facilitators to retention across men with varying levels of ART engagement. Interviews with men who never returned to care may provide more insights into insurmountable barriers to retention. Moreover, it is possible that some retention respondents had initiated care ≥ 14 days after diagnosis, meaning they also met inclusion criteria for initiation respondents; however, we did not have these data available and could not explore this potential overlap. We are also only able to report on the facilitators that participants experienced across the cascade; we did not ask whether participants might benefit from any specific, additional support services that they were not exposed to. In addition, the data are from 2016 to 2017, and new policies may change how men currently experience ART services. Since data collection, Malawi MOH has adopted six-month dispensing for stable clients (i.e., clients on ART ≥ 6 months and virally suppressed). However, programmatic data show that only 42% of eligible clients actually get six-month MMD [50], meaning that the new policy likely does not change most of men’s experiences with ART. All data were collected under universal treatment policies and the implementation of same-day treatment initiation. Finally, findings may not be applicable to settings outside Malawi.

## Conclusion

Men in Malawi who struggle to engage in HIV services experience similar barriers and facilitators for both ART initiation and retention. Holistic interventions that incorporate convenient, private, and client-centered service delivery strategies, as well as male role models and peer support, could improve men’s engagement across the treatment cascade.

## Data Availability

This is a secondary analysis of pre-existing data. Availability of primary data is described in the primary papers published (see citations in main paper).
